# An Island Rash: A Case Study of Exercise-Induced Vasculitis

**DOI:** 10.7759/cureus.88909

**Published:** 2025-07-28

**Authors:** Manuel Ferreira Veloso, Catarina Alves da Silva, Joana Ferreira Vieira

**Affiliations:** 1 Family Medicine, Unidade de Saúde Familiar São Bento, Unidade Local de Saúde Santo António, Porto, PRT; 2 Dermatology, Hospital da Luz Arrábida, Vila Nova de Gaia, PRT; 3 Family Medicine, Unidade de Saúde Familiar São Pedro da Cova, Unidade Local de Saúde Santo António, Porto, PRT

**Keywords:** cutaneous vasculitis, heat-related illness, post-exercise, self limiting, small vessel vasculitis

## Abstract

Exercise-induced vasculitis, also known as the Disney rash or Golfer's vasculitis, is a benign, self-limiting, cutaneous small vessel vasculitis that can develop following prolonged physical activity, especially in hot weather. A 65-year-old female teacher with obesity, diabetes, and dyslipidemia contacted her family physician via email regarding a one-day history of a bilateral lower limb rash. Her medications included metformin and atorvastatin. While vacationing in Madeira, she developed an erythematous, petechial rash above the ankles, circumferentially distributed, following prolonged walks in hot, humid conditions. A telephone consultation was scheduled for further assessment. The lesions, which spared sock-covered areas, worsened over the next 48 hours and were associated with pruritus, mild edema, and warmth. She denied systemic symptoms, recent dietary or medication changes, insect bites, exposure to new hygiene products, and other irritants or allergens. A similar episode had occurred the previous year while on vacation. Bilastine provided no relief. Treatment with mometasone cream was prescribed twice daily. Additionally, the use of compression stockings, leg elevation, and reduced physical activity was advised. A dermatology consultation, requested by the family physician, confirmed a presumptive diagnosis of exercise-induced vasculitis. The rash resolved completely by the 10-day follow-up consultation in primary care. Exercise-induced vasculitis remains underrecognized despite its relative frequency. It commonly affects women over 50 after extended exertion in warm climates. While self-limiting and resolving within 10 to 14 days, treatment is mainly supportive. When triggering conditions persist, relapses are frequent. Family physicians should be aware of the characteristic presentation of exercise-induced vasculitis, as this allows early diagnosis and prevents unnecessary interventions.

## Introduction

Vasculitis can be classified as small, medium, or large according to the caliber of the affected dominant vessels. Cutaneous vasculitis involves an inflammation of the blood vessels within the skin. This disrupts normal blood flow and can cause tissue damage due to reduced oxygen supply. Similarly, capillaritis is a skin disorder marked by inflammation of the small vessels in the upper dermis, leading to the red blood cells leaking into surrounding tissues, and resulting in characteristic pigmentation [1,2]. These conditions include multiple subtypes, with some overlap between the different forms. To differentiate and confirm the exact diagnosis, a skin biopsy is necessary [2,3].

Exercise-induced vasculitis (EIV) is a benign, self-limiting, cutaneous small vessel vasculitis that can develop following prolonged physical activity, especially in hot weather [4]. Although it is a common and clinically relevant condition, it is rarely recognized and diagnosed and often mistaken for other conditions [1,5], especially in primary care and emergency room settings.

This condition is also known as Disney rash or Golfer’s vasculitis, since many people notice this rash after spending long days walking around large theme parks such as Disney World or after spending a long time walking on golf courses, especially during hot weather and humid conditions.

It predominantly affects females over 50 years of age [6,7] and manifests as an erythematous exanthema or pigmented purpuric papules on the lower limbs, mainly the ankles and lower legs, and may be associated with pruritus, burning, and lower limb edema [4]. The lesions may also present as slightly elevated papules [2].

Some usual activities that can trigger EIV include walking, jogging, and running (especially long-distance running), hiking and climbing, step aerobics, bodybuilding, golf, and swimming [5]. These kinds of activities, associated with physical exertion and hot and humid weather conditions, are very common while travelling, especially to island and tropic destinations, as in the setting of this case. 

## Case presentation

**Figure 1:**
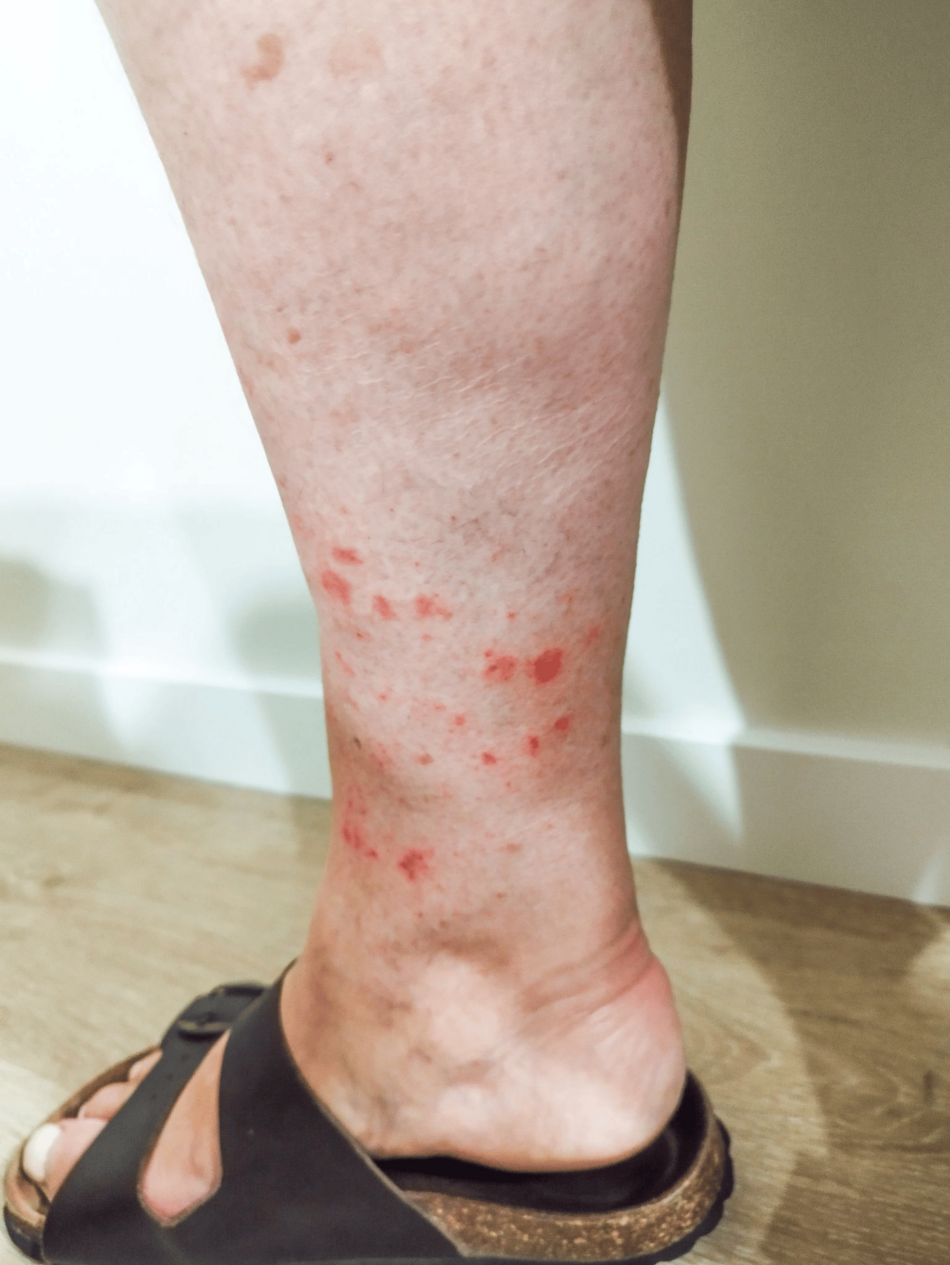
Erythematous and purpuric macules and papules on the leg

A 65-year-old female teacher with a history of obesity, diabetes mellitus, dyslipidemia, and Wolff-Parkinson-White syndrome contacted her family physician via email regarding a skin rash (Figure 1) on both lower limbs that had appeared the previous day.

Her current medications included metformin and atorvastatin. The patient maintained a bi-weekly exercise regimen and had no history of tobacco or alcohol use. There was no relevant dermatological or rheumatological history. Her past surgical history included lipoma excision in 2016. Preventive health measures, including vaccinations, were up to date at the time of presentation.

The patient was on vacation in the Madeira Archipelago and associated the onset of the rash with high temperatures and humidity. She reported taking long daily walks, hiking, and frequent contact with water, with the rash sparing sock-covered areas of the lower limbs.

**Figure 2:**
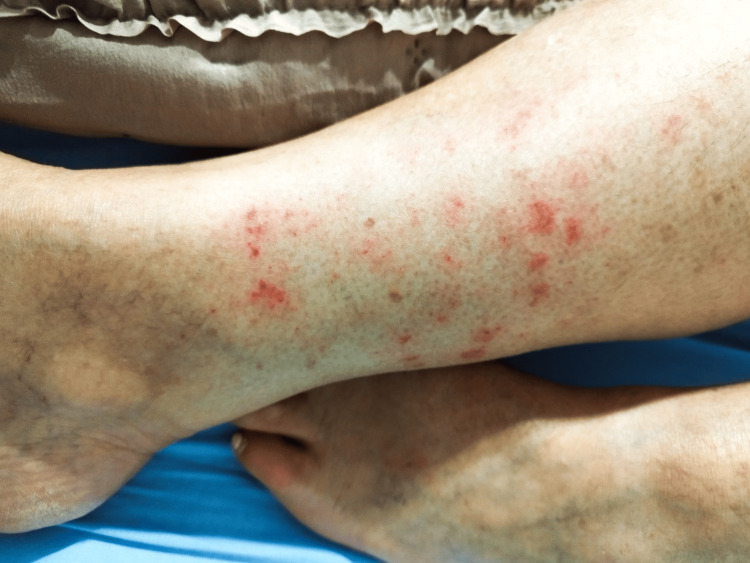
Erythematous and purpuric macules and papules on the legs (48 hours after initial presentation)

The family doctor scheduled a telephone consultation for further assessment. Based on the photographs sent by the patient, and the physician’s visual analysis, the doctor documented a circumferential erythematous exanthema on both lower limbs above the ankles, characterized by petechial lesions measuring approximately 1 cm in diameter. The rash spanned an area of approximately 10 cm in height. The lesions progressively worsened over 48 hours and were accompanied by intense pruritus, mild edema, and a burning sensation (Figure 2).

The patient denied systemic symptoms such as fever, respiratory or gastrointestinal complaints, or urinary changes. She also denied any changes in medication, personal hygiene products, cosmetics, or detergents, contact with other irritants or allergens, insect bites, as well as excessive alcohol/tobacco consumption or dietary risks (e.g., raw meat or seafood). She self-medicated with bilastine (20 mg daily) without significant improvement.

She recalled a similar episode the previous year while on vacation at a warm destination, which she had attributed to insect bites at that time.

Given the clinical presentation and the inability to conduct an in-person examination, the family physician requested a dermatology teleconsultation for further evaluation. The dermatology team, despite the absence of a direct physical examination, after reviewing the case, made a presumptive diagnosis of EIV based on the history and clinical findings. No further investigations were conducted. 

Treatment was then initiated. The patient was prescribed mometasone furoate 0.1% cream to be applied twice daily for one week. Additionally, she was advised to wear compression stockings, elevate her legs, and avoid heat and excessive physical activity, such as intense walks.

**Figure 3:**
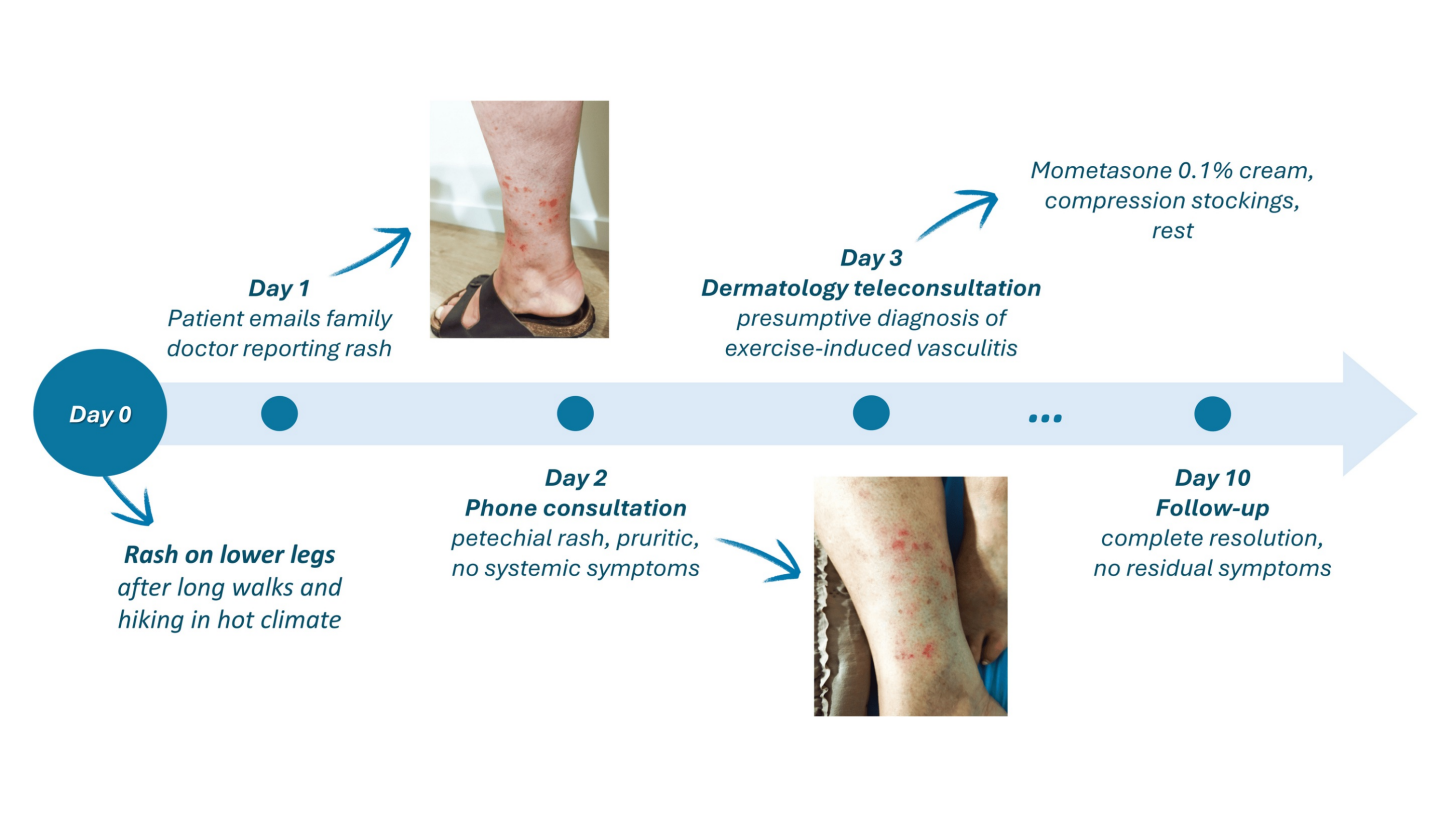
Chronological sequence of events and management actions Image jointly created by the authors in Microsoft Powerpoint (Microsoft Corp., Redmond, WA, US).

At a 10-day follow-up with the family physician, the erythematous lesions had completely resolved, and the patient reported no residual symptoms (Figure 3).

## Discussion

EIV represents a relatively common dermatological entity that remains significantly underdiagnosed in clinical practice [7]. This condition constitutes a stereotypical clinical entity and manifests with a characteristic presentation following prolonged exercise or unusual physical activity, such as a long walk, running, or hiking, particularly in warm climates [5], but also after other activities such as dancing, cycling, or intensive cleaning [7]. In the case under discussion, the symptoms emerged after prolonged ambulatory activity, such as long daily walks and hiking, in the temperate, sun-drenched environment of Madeira Island.

This pathology presents as erythematous or purpuric papules or plaques on the lower legs, notably sparing areas covered by compression garments [2,4]. This characteristic distribution pattern is a key diagnostic feature. Symptoms include itching, pain, and a burning sensation, often accompanied by lower limb edema [1]. EIV appears only after intense physical activities, unlike other idiopathic recurrent cutaneous vasculitis. Heat plays an important role in the occurrence of this condition [4,7]. All these findings were present in the current case. The hot and humid microclimate of Madeira Island contributed to the onset of symptoms, a factor worth considering in similar island or tropical settings.

Differential diagnosis is essential to distinguish EIV from other dermatological and systemic conditions presenting with similar lower limb lesions. Key differentials include leukocytoclastic vasculitis, urticarial vasculitis, systemic immune-mediated vasculitis, contact dermatitis, stasis dermatitis, and cellulitis.

Leukocytoclastic vasculitis typically manifests as palpable purpura accompanied by systemic symptoms such as fever or arthralgia and laboratory abnormalities, none of which were present in this case. It often follows drug exposure, infection, or systemic disease [8], which were absent here. Urticarial vasculitis presents as urticarial plaques, persisting over 24 hours, and frequently resolving with pigmentation or bruising. Systemic symptoms and recurrence are common [9]. In contrast, EIV usually arises after intense exercise, lacks systemic involvement, and resolves rapidly with supportive care, as illustrated in this case. Immune-mediated systemic vasculitis, by definition, involves multiple organs with corresponding symptoms and abnormal laboratory findings such as renal impairment [6]. These are not features of EIV.

Contact dermatitis presents with erythema and pruritus but is characteristically well-demarcated and linked to allergen or irritant exposure, which the patient denied. Moreover, the sparing of sock-covered areas argues against a contact etiology [10]. Stasis dermatitis, more common in individuals with chronic venous insufficiency, is typically chronic and localized near the medial malleolus [11]. This was not observed in this case. Cellulitis generally shows rapid progression, pain, ill-defined margins, and systemic signs [12], unlike the acute, bilateral, circumferential lower leg lesions seen in EIV.

Taken together, the acute onset of bilateral lower leg lesions following prolonged walking in hot, humid conditions, spontaneous resolution within days, sparing of sock-covered skin, and recurrence under similar circumstances strongly support the diagnosis of EIV [4,6]. This condition also demonstrates a predilection for females (62.1%) over 50 years of age [1,5-7], and our patient fits this typical demographic. While it typically resolves spontaneously within 10 to 14 days, as represented in this case, management may include topical corticosteroids, non-steroidal anti-inflammatory drugs (NSAIDs), compression stockings, lower limb elevation, lymphatic drainage, among others, to alleviate symptoms and promote recovery [4,6].

In this case, the family physician requested a dermatology teleconsultation. Despite the absence of a direct physical examination, the typical distribution pattern, temporal association with exertion in a warm environment, recurrence, and epidemiological patterns supported the presumptive diagnosis. No laboratory tests or biopsies were deemed necessary, as the clinical picture was typical and self-limited.

If systemic symptoms had been present, further testing would have been performed. Laboratory evaluation could have included complete blood count with differential and platelets, comprehensive metabolic panel and urinalysis with microscopy, HIV, hepatitis B and C serologies, serum complement levels, antinuclear antibody (ANA), anti-neutrophil cytoplasmic antibody (ANCA), Anti-double-stranded DNA (anti-dsDNA), anti-Ro, anti-La, anti-ribonucleoprotein (RNP), and anti-Smith antibodies, along with rheumatoid factor, serum cryoglobulins, and biopsy for direct immunofluorescence [13].

## Conclusions

EIV is a distinct dermatological condition, frequently overlooked in clinical practice, that predominantly affects women over the age of 50 and is closely linked to prolonged physical activity in warm environments. Its hallmark features - bilateral erythematous or purpuric lesions on the lower legs that spare areas protected by compression - are key to diagnosis, especially when accompanied by pruritus, burning, and edema following exertion. Although EIV is generally self-limiting and resolves within two weeks, symptomatic management with topical corticosteroids, NSAIDs, compression therapy, and limb elevation can provide relief and expedite recovery.

This case reinforces the importance of considering EIV in patients presenting with lower limb purpura following physical exertion in warm environments, especially when lesions spare compression-covered areas. Early recognition can prevent unnecessary investigations and improve patient reassurance. Therefore, family physicians and emergency doctors should be aware of this condition to ensure timely recognition and management.
